# Correspondence: Prostatic sarcoma after treatment for rectal cancer

**DOI:** 10.1186/1477-7819-6-20

**Published:** 2008-02-17

**Authors:** Noel J Aherne, Charles M Gillham

**Affiliations:** 1 Department of Radiation Oncology, St. Luke's Hospital, Dublin 6, Ireland

## 

Dear Sirs,

The authors Abbas SM, and Hill AG [1] outline their experience of a case of prostatic sarcoma occurring four years post neoadjuvant radiation therapy for primary mid rectal adenocarcinoma. They correctly state that as this second malignancy occurred within the radiation field it meets the criteria for a radiation – induced second malignancy. However, it has been noted that while there is no established dose response relationship for sarcoma that they are generally felt to occur within the high dose region. A dose of 4500 cGy seems unlikely to lead to sarcoma formation within such a short time frame.

While post radiation sarcomas have commonly been described in the pelvis they more commonly follow high dose 3 Dimensional Conformal Radiation therapy (3DCRT) with or without intracavitary brachytherapy (doses > 7000 cGy) in gynecological cancers, or in combination with interstitial seed brachytherapy (doses > 1000 cGy) in prostatic malignancy. In contrast, primary prostatic sarcoma is a well recognized clinical entity with over 50 cases recorded in the literature [2-4].

While the author's assertion that this is a radiation induced second malignancy is supported by the temporal association, radiobiologically it is not. However, this case is a salient reminder of the significant risks associated with all forms of cancer therapy.

## Competing interests

The author(s) declare that they have no competing interests.

## Authors' contributions

NJA: Conception and design, editing of manuscript.

CMG: preparation of draft manuscript.

All authors read and approved final manuscript.
